# Associations of light exposure patterns with sleep among Dutch children: The ABCD cohort study

**DOI:** 10.1111/jsr.14184

**Published:** 2024-02-27

**Authors:** Magdalini Stefanopoulou, Naomi Ruhé, Lützen Portengen, Luuk van Wel, Tanja G. M. Vrijkotte, Roel Vermeulen, Anke Huss

**Affiliations:** ^1^ Institute for Risk Assessment Sciences Utrecht University Utrecht The Netherlands; ^2^ Department of Public and Occupational Health, Amsterdam Public Health Research Institute, Amsterdam UMC University of Amsterdam Amsterdam The Netherlands; ^3^ Julius Centre for Health Sciences and Primary Care University Medical Centre Utrecht Utrecht The Netherlands

**Keywords:** accelerometry, children, light exposure, light meter, sleep duration, sleep onset

## Abstract

Light exposure affects the circadian system and consequently can affect sleep quality. Only few studies examined this relationship in children. We evaluated associations between light exposure patterns and sleep metrics in children. We measured the sleep parameters of 247 Dutch children, aged between 11 and 13 years and recruited from the ABCD cohort, using actigraphy and sleep records for 7 consecutive nights. Personal light exposures were measured with a light meter during the whole day and night. We applied generalized mixed‐effects regression models, adjusted for possible confounders, to evaluate the associations of light exposure patterns on sleep duration, sleep efficiency and sleep‐onset delay. In the models mutually adjusted for potential confounders, we found the amount of hours between the first time of bright light in the morning and going to sleep and the duration of bright light to be significantly associated with decreased sleep duration (in min; β: −2.02 [95% confidence interval: −3.84, −0.25], β: −8.39 [95% confidence interval: −16.70, −0.07], respectively) and with shorter sleep‐onset delay (odds ratio: 0.88 [95% confidence interval: 0.80, 0.97], odds ratio: 0.40 [95% confidence interval: 0.19, 0.87], respectively). Increased light intensities at night were associated with decreased sleep duration (T2 β: −8.54 [95% confidence interval: −16.88, −0.20], T3 β: −14.83 [95% confidence interval: −28.04, −1.62]), while increased light intensities before going to bed were associated with prolonged sleep onset (odds ratio: 4.02 [95% confidence interval: 2.09, 7.73]). These findings further suggest that children may be able to influence their sleep quality by influencing the light exposure patterns during day and night.

## 1 INTRODUCTION

Healthy sleep is considered important for maintaining physical and mental health (Liu et al., 2016), but sleep problems are becoming increasingly prevalent worldwide (Castro‐Diehl et al., 2018). Currently, approximately 9%–15% of adults have been diagnosed with chronic sleep problems (Akbarpour et al., 2022), while about 4% of children face sleep disorders (Chen et al., 2021). Sleep problems have been associated with, for example, obesity (Mercy et al., 2022), cardiovascular disease (Huang et al., 2022), cancer (Peeri et al., 2022), and depressive or anxiety symptoms (Joo et al., 2022; Oakes et al., 2022). Several risk factors have been associated with sleep problems, like chronic stress, air pollutants and blue light exposure (Cajochen et al., 2005; Hall et al., 2015; Wang et al., 2022a). However, further research is needed to identify additional modifiable risk factors of sleep problems that can inform preventive strategies.

Light exposure patterns have changed substantially over the last century due to rapid urbanization and population growth, resulting in humans spending most of their daytime indoors exposed to low levels of natural light, while spending their nighttime exposed to higher intensities of artificial light (Bonmati‐Carrion et al., 2014). There are two major ways how light exposure can affect sleep quality. First by extending daylength into the night due to electric lighting, which creates favourable conditions for recreation and leisure activities, and subsequently affects the quality or duration of sleep (Stevens, 2009). Secondly, by disturbing the circadian rhythm and inhibiting secretion of melatonin, as light is a strong environmental cue of the circadian rhythm (Walker 2nd et al., 2022). Therefore, a natural light exposure pattern that consists of both a low light intensity at night and a high light intensity during the day has been described as relevant for the proper activation of the circadian system (Bonmati‐Carrion et al., 2014).

Previous studies investigated relationships between light exposure and sleep problems (Cho et al., 2015; Paksarian et al., 2020). Observational and experimental studies indicated light exposure in the hour before or during sleep can lead to a delayed sleep onset and reduced sleep quality (Cho et al., 2015). Yet, little is known about the possible effects of the timing and duration of bright daylight on sleep. Additionally, only few studies have been performed in children. Two studies showed associations between outdoor artificial light at night (ALAN) and sleep patterns or sleep duration in adolescents (Paksarian et al., 2020; Vollmer et al., 2012), while one study suggested sleep disorders were more prevalent in children residing in areas with higher levels of outdoor ALAN, with stronger associations in children younger than 12 years old (Huss et al., 2019; Wang et al., 2022b). Pupil size and light transmission rate of the crystalline lens decreases with aging and, therefore, children may be more sensitive to effects of light exposure (Crowley et al., 2015; Higuchi et al., 2014). Hence, it is important to better understand light exposure effects on sleep patterns on this younger group to consider for optimal light environments for children.

To address this research gap, we investigated the associations between light exposure patterns during the whole day and the sleep metrics duration, efficiency and sleep‐onset delay in a group of Dutch children through objective measurements.

## 2 METHODS

### 2.1 Study population

We included 247 children from the Netherlands aged between 11 and 13 years into our study. Children were recruited from the ABCD prospective cohort study, which investigates the relationship between maternal lifestyle and psychosocial factors during pregnancy to several health outcomes in children. The study design of the ABCD study has been described in detail before (van Eijsden et al., 2011). Briefly, 8266 pregnant women were enrolled between January 2003 and March 2004 during their first prenatal visit to an obstetric care provider, and they were asked to fill out a baseline questionnaire. When the children of the study reached the ages of 3 months, 5, 11 and 16 years, follow‐ups were performed. In the 11‐year follow‐up, the 3027 included children were asked to participate in a campaign about light and physical activity measurements. In total, 786 participants agreed, and after contacting them 368 (47%) were willing to participate. Two‐hundred and forty‐seven participants willing to participate in the campaign during a school week formed our study population and can be seen as a volunteer sample within the ABCD cohort study.

The ABCD cohort has been approved by the Central Committee on Research involving Human Subjects in the Netherlands, the medical ethical committees of the participating hospitals, and the Registration Committee of the Municipality of Amsterdam.

### 2.2 Light exposure assessment

Personal light exposures were assessed between December 2015 and July 2017 by asking the study participants to wear a light meter (LightWatcher; Wolf Technologieberatung) for 1 week. The LightWatcher is a small (1 × 2 × 5 cm) and lightweight (~12 g) device, which can be set to high logging intervals (e.g. every 2–10 s) and can measure light intensity in units of lux. In our study, the LightWatchers were set to measure every 2 s and to log these measurements as 10‐s averages in order to reserve battery, with a resolution of 0.1 lux and a limit of quantification of 0.01 lux. The devices were checked and calibrated before and during the study.

Participants were asked to wear the LightWatcher for 7 days (8 nights), to capture both weekday and weekend sleep patterns. The children wore the light meter as a pendant during the day, as this is a non‐intrusive place to wear the meters, and they were asked to put the device on their nightstand when they went to sleep. After the 7 days, the LightWatchers were sent back to Utrecht University for data download and processing (Huss et al., 2019).

We evaluated the following light parameters in this study: (1) timing of bright light in the morning, which was measured as the number of hours between the first time of bright light in the morning and going to sleep; (2) timing of bright light in the afternoon, which was measured as the amount of hours between the last time of bright light in the afternoon and going to sleep; (3) bright light duration, which was the duration of bright daylight exposure exceeding 1000 lux (hr); (4) average light intensity in the hour before going to bed (lux); and (5) average light intensity during the darkest part of the night (00:12 hours–03:10 hours; lux; Huss et al., 2019).

### 2.3 Sleep assessment

To assess sleep objectively, participants were asked to wear wrist‐accelerometers (Actigraphs) on the non‐dominant wrist during a whole week to measure sleep parameters including sleep duration, sleep latency and sleep efficiency. The GT3X+ Actigraph Accelerometer is an omnidirectional accelerometer that is sensitive to movement in all directions. It is relatively small (5.1 × 3.8 × 1.5 cm) and lightweight (43 g). It is a non‐intrusive method that can be used for assessing sleep patterns objectively. Children were also asked to record in a diary when they did not wear the sensors, when they went to bed and when they got up. Recordings of activity–rest patterns were calculated based on the outputs from three accelerometers with a frequency limit of 30 Hz. Together with the LightWatchers, Actigraphs and the diaries were sent back to the study centre after 1 week of measurements.

In this study, we used three sleep outcomes: (1) sleep duration (measured in minutes per night), which was defined as the accumulated nocturnal sustained inactivity bouts by the Actigraph; (2) sleep efficiency, which was the sleep duration divided by the total time in bed (difference between onset and waking time, expressed as percentage), both measured by the Actigraph; (3) sleep‐onset delay (measured in minutes), which was the difference between sleep onset as estimated by the Actigraph and the time children went to bed as recorded in the sleep logs.

### 2.4 Statistical analysis

Descriptive statistics were applied to describe the study population socio‐demographic characteristics, and differences of sleep and light parameters in the total population and in subgroups. To assess the effect of bright light duration, light intensity in the hour before bed and light intensity at night, we categorized the measurements into three groups: the reference group, which covered the lowest values until the median; the low exposed group, which were the values from 50% to 90%; and the high exposed group, which represented the highest 10% of the measurements.

We calculated the effect of light exposure parameters on sleep outcomes by using mixed‐effects regression models for the repeated measures on 7 days. We included subject ID as a random intercept in all models to account for between‐subject variation. After removing outliers from outcome measures, which were defined as the values exceeding three times the standard deviation (*n* = 20 observations), sleep duration followed a normal distribution. Therefore, associations of light exposure parameters with sleep duration were based on linear mixed‐effects models. Sleep efficiency followed a left skewed distribution, and therefore a beta regression was used. Because sleep‐onset delay did not follow a normal distribution, it was dichotomized, and logistic mixed‐effects models were used. The lowest 80% of values were defined as the “controls” (0–58 min), and the highest 20% values were defined as the “cases” (59–213 min).

The models were minimally, fully and mutually adjusted for potential confounders. Minimally adjusted models were adjusted only for age and sex. Fully adjusted models additionally accounted for maternal education (low/high), weekend night (yes/no) and season (April–September/October–March) in which the measurements were done, due to previous observations of higher sleep quality during summer than winter (Figueiro & Rea, 2016). The mutually adjusted models additionally accounted for the other exposure metrics. We also estimated trends across exposure groups, using the midpoints (means) of our exposure categories for this calculation. Generalized linear and logistic mixed‐effects models were built using the “lme4” R package (Bates et al., 2015), and beta regression models were built using the “glmmTMB” R package (Brooks et al., 2017). The results obtained by the models are presented as beta estimates for sleep duration, odds ratios (ORs) for sleep‐onset delay and exponentiated coefficients for sleep efficiency with their corresponding confidence intervals (95% CI).

We also performed sensitivity analyses. First, we investigated potential interaction variables. Social jetlag – caused by a misalignment between the social time and the biological clock, due to different bed times on weekend and weekdays (Bonmati‐Carrion et al., 2014) – and weekend night (yes/no) could have an interaction with light exposure parameters. Social jetlag was calculated as the duration measured in hours between the midpoint of sleep duration on weekends minus the midpoint of sleep duration on weekdays, and it was dichotomized at the 80th percentile into low (−3.21–1.65 hr) and high (1.65–3.33 hr) social jetlag. The potential interaction between bright light duration and light intensity in the hour before sleep was also assessed; interactions were assessed using fully adjusted models. Secondly, we repeated our analysis for sleep efficiency based on sleep log entries: sleep efficiency can be calculated based on measurements taken by the Actigraph, or alternatively by dividing sleep duration measured by the Actigraph by the total time in bed according to the sleep log. Because scientific literature does not provide a clear preference for the way of calculating sleep efficiency (Smith et al., 2018) and as Actigraphs provide an objective measurement, we defined sleep efficiency based on measurements taken by the Actigraph as our primary analysis and sleep log‐based sleep efficiency as our sensitivity analysis to assess if this method yielded different results. R (version 1.2.5019) was used for all calculations.

## 3 RESULTS

Of the 247 children in our study, 110 were boys while 137 were girls. Demographic characteristics are shown in Table 1. The mean sleep duration was 466.70 min per night (SD = 55.72 min per night), and the medians of sleep efficiency measured by Actigraph and sleep‐onset delay were 84.4% (interquartile range [IQR] = 79.5%–88.6%) and 26.4 min (IQR = 8.9–51.3 min), respectively. Table S1 shows the distribution of sleep outcomes across sociodemographic characteristics of the study group. The mean time in bed as measured by sleep logs was 561 min per night (SD = 66.62 min per night), and the median of sleep efficiency measured by sleep logs was 76.4% (IQR = 70.5%–81.4%). The mean for timing of bright light in the morning was 11.5 hr (SD = 3.0), while the mean for timing of bright light in the afternoon was 3.8 hr (SD = 2.5). The mean duration of bright light was 1.28 hr (SD = 1.27). The mean intensity of the light before going to bed was 74.64 lux (SD = 432.57), while the mean intensity of light when in bed was 0.29 lux (SD = 1.66). Variations in sleep routines and light conditions influenced by factors such as the day of the week (weekday versus weekend) and seasonal changes (winter or summer) can potentially explain the low minimum values. For instance, a minimum value of zero hours for “bright light duration” indicates a complete lack of daylight exposure during the day, which can occur during winter with limited daylight or during a day spent indoors (Table 2).

**TABLE 1:** Demographic characteristics of 247 study participants.

(Sub)group	Participants
Sex	
Boys	110
Girls	137
Maternal education	
Low	48
High	199
Age (years)	
11	80
12	123
13	44
Season	
Winter	103
Summer	144

*Note*: Maternal education: low (higher and lower secondary school); high (university degree). Season: summer (April until September); winter (October until March).

**TABLE 2:** Overview of the sleep and light exposure parameters.

	Mean	SD	Median	IQR	Min	Max
Sleep outcomes						
Sleep duration (min per night)	466.70	55.72	467.40	432.30–501.70	285.30	630.20
Time in bed (min per night)	561.40	66.62	557.90	520.2201–600.3	175.90	977.80
Sleep efficiency (by Actigraph) (%)	83.13	8.18	84.38	79.52–88.60	20.03	99.47
Sleep efficiency (by sleep logs) (%)	75.25	9.03	76.44	70.51–81.38	20.03	98.98
Sleep‐onset delay (min)	35.04	34.89	26.42	8.92–51.33	< 1	213.00
Light exposure metrics						
Timing of bright light in the morning (hr before sleep)^a^	11.47	2.99	12.30	10.40–13.35	0.35	17.07
Timing of bright light in the afternoon (hr before sleep)^a^	3.77	2.46	3.27	1.93–5.22	< 0.1	15.82
Duration of bright light (hr)	1.28	1.27	0.93	0.26–1.90	0.00	9.77
Average light intensity before going to bed (lux)	75.37	431.62	12.07	4.97–27.81	< 0.01	10,484.93
Average light intensity when being in bed (lux)	0.29	1.66	0.07	0.02–0.16	< 0.01	28.82

*Note*: Sleep duration (min per night): (Actigraph); time in bed (min per night): (sleep logs): diff. wake up – going to bed; sleep efficiency (%): (Actigraph): sleep duration/total time in bed; sleep efficiency (%) (sleep logs): sleep duration (Actigraph)/total time in bed (sleep logs); sleep‐onset delay (min): (diff. Actigraph; sleep logs); timing of bright light in the morning: hours between first time of bright light in the morning and going to sleep; timing of bright light in the afternoon: hours between last time of bright light in the afternoon and going to sleep; bright light duration: hours of daylight exposure (>1000 lux); average light intensity before going to bed: average lux in hours before going to bed; average light intensity when in bed: average lux during the darkest part of the night (00:12 hours–03:10 hours).

Abbreviation: IQR, interquartile range.

^a^ The variables timing of bright light in the morning and timing for bright light in the afternoon were determined solely for participants who experienced bright light exposure on the respective days.

Table 3 shows the results of mutually adjusted mixed‐effects regression analyses on sleep duration, sleep efficiency and sleep‐onset delay. Tables S2–S4 show results of the minimally and fully adjusted mixed‐effects regression. Sleep duration was slightly shorter in the mutually adjusted model in children with earlier bright light exposure and with longer duration of bright light. An increase in light intensities at night was associated with a decrease in sleep duration. At light intensities at night in the low exposure group (0.07–0.4 lux), children slept on average 9 min less; and in the high exposure group (>0.4 lux), sleep duration decreased by approximately 15 min. Confounder adjustment did not materially affect estimates (Table S2). The timing of morning bright light as well as longer bright light duration during the day was associated with faster sleep onset, with a trend (*p*‐trend = 0.02) across exposure categories. Children with high light exposure (>80 lux) in the hour before they went to bed had an increased OR for being in the late sleep‐onset delay group compared with those exposed to < 12 lux, with a clear trend (*p*‐trend <0.001) across exposure categories. For sleep efficiency, exponentiated coefficients were close to unity, with a possible exception of light exposure during the night (*p*‐trend = 0.06).

**TABLE 3:** Results of mixed‐effects regression analysis for sleep duration, sleep efficiency and sleep‐onset delay, mutually adjusted for age, sex, maternal education, weekend night, season and other light exposure metrics.

	Sleep duration (min per night) β (95% CI)	Sleep efficiency (%) exp coefficients (95% CI)	Sleep‐onset delay (min) OR (95%CI)
Timing bright light morning (hr before sleep)	**−2.02 [−3.84, −0.25]**	1.01 [0.99, 1.03]	**0.88 [0.80, 0.97]**
Timing bright light afternoon (hr before sleep)	−1.13 [−2.99, 0.72]	1.02 [1.00, 1.04]	1.04 [0.94, 1.15]
Duration of bright light (hr)			
Below median: < 0.9	Reference group	Reference group	Reference group
Median–90th perc.: 0.9–3.0	**−8.39 [−16.70, −0.07]**	0.97 [0.90, 1.05]	0.98 [0.62, 1.57]
>90th percentile: >3.0	−5.03 [−17.58, 7.52]	1.00 [0.89, 1.13]	**0.40 [0.19, 0.87]**
*p*‐trend	*0.43*	*0.90*	***0.02***
Average light intensity (lux) before going to bed			
Below median: < 12	Reference group	Reference group	Reference group
Median–90th perc.: 12–79	1.01 [−6.38, 8.41]	0.93 [0.86, 1.00]	1.27 [0.83, 1.94]
>90th percentile: >80	3.95 [−8.83, 16.33]	0.96 [0.86, 1.09]	**4.02 [2.09, 7.73]**
*p*‐trend	*0.31*	*0.85*	***< 0.001***
Average light intensity (lux) when in bed			
Below median: < 0.07	Reference group	Reference group	Reference group
Median–90th perc.: 0.07–0.4	**−8.54 [−16.88, −0.20]**	0.95 [0.88, 1.04]	1.03 [0.65, 1.62]
>90th percentile: >0.4	**−14.83 [−28.04, −1.62]**	0.88 [0.77, 1.00]	1.30 [0.65, 2.62]
*p*‐trend	*0.07*	*0.06*	*0.50*

*Note*: Sleep duration (min per night): (Actigraph); sleep efficiency (%): (Actigraph): sleep duration/total time in bed; sleep efficiency (%) (sleep logs): sleep duration (Actigraph)/total time in bed (sleep logs); sleep‐onset delay (min): (diff. Actigraph; sleep logs); timing of bright light in the morning: hours between first time of bright light in the morning and going to sleep; timing of bright light in the afternoon: hours between last time of bright light in the afternoon and going to sleep; bright light duration: hours of daylight exposure (>1000 lux); average light intensity before going to bed: average lux in hours before going to bed; average light intensity when in bed: average lux during the darkest part of the night (00:12 hours–03:10 hours). Values in bold indicate statistical significance.

Abbreviations: CI, confidence interval; OR, odds ratio.

Our sensitivity analysis of bright light duration and the light intensity in the hour before bed revealed no significant interactions for all three outcomes. Furthermore, adding social jetlag or weekend night (yes/no) as interaction term did not lead to a significantly improved fit of the model for any of the three outcomes. When we repeated the analysis for sleep efficiency, using the data from the sleep logs, results remained similar to the initial analysis (Table S5).

## 4 DISCUSSION

In this study, we investigated associations between measured light exposure patterns during a whole week and sleep duration, sleep efficiency and sleep‐onset delay in a population of Dutch children. Our results showed that time between the first morning bright light and going to bed, and bright light duration were associated with decreased sleep duration and a quicker sleep onset. Increased light intensities at night were associated with shorter sleep duration, while higher light intensities before going to bed were associated with a longer sleep‐onset delay. Associations between light intensity and sleep duration and between bright light duration, light density and sleep‐onset delay were not materially affected when adjusted for a range of potential confounders.

These findings should be interpreted in the context of the study's strengths and limitations. Much of the evidence in the relationship between light exposure patterns and sleep health comes from experimental studies. Experimental studies are considered as the gold‐standard approach to investigate how light affects sleep, as exposure conditions can be standardized, and other factors can be controlled. However, experimental studies may not realistically reflect real‐life situations. For instance, the spatial and temporal heterogeneity of light exposures in urban environments or home may not be captured by the homogenous laboratory light. Additionally, in the laboratory, an individual's sleeping behaviour might differ from their usual sleeping behaviour (Aulsebrook et al., 2018). Therefore, observational designs that are suitable for studying larger populations are also valuable to evaluate real‐life situations. Strengths of our study include the large sample size and the repeated measurements during a full week, so that both weekdays and weekends during a normal school week were included. Additionally, we used a light measurement device that was small and lightweight, so that personal exposures during the day and bedroom exposures during the night could be measured objectively. Until now, several studies used ALAN by satellite measurements or by self‐reports of sleeping room brightness to assess light exposure. However, outdoor ALAN from satellites does not accurately reflect indoor evening or nighttime light exposure (Huss et al., 2019; Xu et al., 2023), while it is unclear to which light levels self‐reports correspond.

In addition to these strengths, the study has limitations. Although actigraphy is a non‐intrusive method for assessing motion from which periods of sleep and wakefulness may be inferred, it is not the gold‐standard like polysomnography (PSG). Over the past two decades the usage of actigraphy has increased. Especially in children it can be an additional valuable methodology for estimating sleep behaviour, because subjective reports of the parents alone may not be very reliable (Meltzer et al., 2012). Numerous studies have documented that actigraphy tends to overestimate total sleep time (Kaplan et al., 2012). However, a validation study in healthy adults showed actigraphy had a 90% concordance with PSG (Jean‐Louis et al., 2001). Light measurements were done using LightWatchers, which were worn as a pendant or placed on a bedside table. This method may have underestimated the actual light exposure at eye level (Figueiro et al., 2013). Our measurements aligned with our expectations. The values of our sleep parameters (Table 2) closely resembled those found in a previous study that objectively measured sleep using actigraphy (Arora et al., 2013). The results of the present study are consistent with previous observational studies in adults showing that exposure to artificial light in the evening or throughout the night can prolong sleep onset (Chang et al., 2015; Cho et al., 2015; Santhi et al., 2012) and decrease total sleep duration (Cho et al., 2016). Others reported exposure to light later in the evening was associated with delayed sleep times, but found no difference in sleep duration (Wams et al., 2017) or no sleep problems were observed (Xu et al., 2023). Inconsistent findings could be explained by multiple factors such as differences in sample size, study design, methods to assess exposures and outcomes, and technical differences of the used equipment. Additionally, timing, duration, intensity and prior exposure of the light exposure can influence the effects of light at night on sleep and alertness. Potential confounders such as increased fatigue associated with exercise may also influence patterns of sleep (Aulsebrook et al., 2018). It is therefore difficult to completely isolate the effects of light exposure from other factors. Our results are also aligned with studies reporting longer sleep durations and later sleep onsets in winter compared with summer (Dunster et al., 2023; Zerbini et al., 2021). Sleep efficiency derived from sleep logs or from actigraphy has been described previously, without a clear preference of one over the other. In contrast to a previous study by Smith et al. (2018), our sensitivity analysis using sleep logs instead of actigraphy data did not reveal deviating results.

## 5 CONCLUSION

Our study findings suggest that not only intensity but also timing and duration of light exposures can influence sleep quality in children and highlight the relevance of the entire 24‐hr pattern of the light–dark profile. Children may be able to influence their sleep quality by influencing the light exposure patterns during day and night. Aligning with a natural light pattern, characterized by increased morning and daytime light and reduced evening and nighttime light, may enhance children's sleep duration and efficiency. Practical recommendations derived from our results could include commuting to school on foot or by bike, incorporating outdoor breaks during school hours, and ensuring a dark sleep environment.

## AUTHOR CONTRIBUTIONS

**Magdalini Stefanopoulou:** Investigation; methodology; formal analysis; writing – original draft. **Naomi Ruhé:** Investigation; methodology; writing – original draft; formal analysis. **Lützen Portengen:** Validation; writing – review and editing. **Luuk van Wel:** Writing – review and editing. **Tanja Vrijkotte GM:** Writing – review and editing. **Roel Vermeulen:** Writing – review and editing. **Anke Huss:** Conceptualization; methodology; project administration; writing – review and editing.

## FUNDING INFORMATION

This work was supported by the European Union (grant 603794) and the Netherlands Organization for Health Research and Development within the Electromagnetic Fields and Health Research program (grants 85600004, 85200001, and 85800001), intramural funds from the Utrecht Exposome Research Hub and the LILLI study 2019–108 funded by ANSES.

## CONFLICT OF INTEREST STATEMENT

All authors confirm that there are no potential conflicts of interest.

## Supporting information


**DATA S1** Supporting Information

## Data Availability

The data that support the findings of this study are available on request from the corresponding author. The data are not publicly available due to privacy or ethical restrictions.
