# Supplementary material for: Associations of light exposure patterns with sleep among Dutch children: The ABCD cohort study
Source: J Sleep Res. 2024 Feb 27;33(6):e14184. doi: 10.1111/jsr.14184 (PMC11596991; doi:10.1111/jsr.14184)
Supplement: Supplementary file 1 — DATA S1 Supporting Information [file JSR-33-e14184-s001.docx]

**Supplementary material**

**Supplementary Table ST1.** Means and medians of sleep duration, sleep efficiency and sleep onset delay per subgroup.

|  | **Sleep duration (min/night) Mean (SD)** | **Sleep efficiency (%) Median (IQR)** | **Sleep onset delay (min) Median (IQR)** |
| --- | --- | --- | --- |
| **All** | 466.70 (55.72) | 84.38 (79.52 - 88.60) | 26.42 (8.92 - 51.33) |
| **Sex** |  |  |  |
| *Boys* | 458.78 (54.32) | 83.50 (78.73 - 87.86) | 28.67 (9.25 - 55.33) |
| *Girls* | 473.12 (56.04) | 85.16 (80.22 - 89.37) | 25.08 (8.48 - 47.06) |
| **Age** |  |  |  |
| *11* | 477.17 (52.71) | 84.51 (79.18 - 88.68) | 30.25 (11.58 - 54.58) |
| *12* | 462.77 (56.25) | 84.94 (78.94 - 88.26) | 25.62 (8.33 - 49.75) |
| *13* | 459.18 (56.97) | 85.24 (80.81 - 89.04) | 23.67 (7.08 - 43.40) |
| **Maternal education** |  |  |  |
| *Low* | 454.83 (62.24) | 83.76 (77.91 - 88.50) | 23.75 (7.33 - 48.17) |
| *High* | 469.7 (53.60) | 84.55 (79.79 - 88.62) | 27.67 (9.06 - 51.83) |
| **Season** |  |  |  |
| *Winter* | 476.34 (54.55) | 84.54 (79.86 - 88.69) | 28.50 (11.04 - 51.83) |
| *Summer* | 459.95 (55.57) | 84.27 (79.18 - 88.44) | 25.00 (6.52 - 51.13) |
| **Social jetlag** |  |  |  |
| *Low* | 467.16 (55.79) | 84.25 (79.58 - 88.51) | 28.63 (9.67 - 53.27) |
| *High* | 465.4 (55.62) | 84.83 (79.23 – 88.93) | 18.88 (5.75 - 40.06) |

**Sleep duration (min/night)**: the accumulated nocturnal sustained inactivity bouts measured by the Actigraph; **Sleep efficiency** **(%)**: the sleep duration divided by the total time in bed, both measured by the Actigraph (difference between onset and waking time); **Sleep onset delay (min)**: the difference between sleep onset as estimated by the Actigraph and estimated by the sleep logs; **Social jetlag:** the duration measured in hours between the midpoint of sleep duration on weekends minus the midpoint of sleep duration on weekdays; **Season:** summer (April until September), winter (October until March); **Maternal education**: low (higher and lower secondary school), higher (university degree)

**Supplementary Table ST2.** The results of the linear mixed effects regression analysis on sleep duration.

|  | Minimally adjusted  β (95% CI) | Sleep duration (min/night)   Fully adjusted  β (95 % CI) | Mutually adjusted  β (95% CI) |
| --- | --- | --- | --- |
| Timing bright light morning (h before sleep) | **-0.91 [-1.77, -0.06]** | -0.72 [-1.59, 0.13] | **-2.02 [-3.84, -0.25]** |
| Timing bright light afternoon (h before sleep) | -0.66 [-1.88, 0.56] | **-1.64 [ -2.96, -0.33]** | -1.13 [-2.99, 0.72] |
| Duration of bright light (hours) |  |  |  |
| **Below median:** < 0.9 | Reference group | Reference group | Reference group |
| **Median-90th perc.:** 0.9 to 3.0 | **-7.05 [-13.05, -1.05]** | -4.55 [-10.71, 1.62] | **-8.39 [-16.70, -0.07]** |
| **>90th percentile:** > 3.0 | -4.30 [-13.83, 5.23] | -2.33 [-12.18, 7.52] | -5.03 [ -17.58, 7.52] |
|  |  |  | *p trend=0.43* |
| Average light intensity (lux) before going to bed |  |  |  |
| **Below median:** < 12 | Reference group | Reference group | Reference group |
| **Median-90th perc.:** 12 to 79 | 1.56 [-3.94, 7.06] | 2.58 [-2.96, 8.11] | 1.01 [-6.38, 8.41] |
| **>90th percentile:** > 80 | 7.32 [-1.77, 16.42] | **9.64 [0.44, 18.85]** | 3.95 [-8.83, 16.33] |
|  |  |  | *p trend= 0.31* |
| Average light intensity (lux) when in bed |  |  |  |
| **Below median:** > 0.07 | Reference group | Reference group | Reference group |
| **Median-90th perc.:** 0.07 to 0.4 | **-8.49 [-16.09, -0.88]** | **-8.18 [-15.69, -0.67]** | **-8.54 [ -16.88, -0.20]** |
| **>90th percentile:** > 0.4 | **-15.33 [-27.37, -3.28]** | **-16.77 [-28.73, -4.82]** | **-14.83 [-28.04, -1.62]** |
|  |  |  | *p trend=0.07* |

**Sleep duration (min/night)**: the accumulated nocturnal sustained inactivity bouts measured by the Actigraph; **The timing of** **bright light in the morning**: the amount of hours between the first time of bright light in the morning and going to sleep; **The timing of bright light in the afternoon**: the amount of hours between the last time of bright light in the afternoon and going to sleep; **Bright light duration**: the duration of bright daylight exposure with a cut-off at 1000 lux (h); **Average light intensity before going to bed**: the average light intensity in the hour before going to bed (lux); **Average light intensity when in bed**: the average light intensity during the darkest part of the night (00:12 – 03:10) (lux); **Minimally adjusted:** Model adjusted for age and sex; **Fully adjusted:** Model adjusted for age, sex, maternal education (low/high), weekend night (yes/no) and season (April-September / October-March); **Mutually adjusted:** Model adjusted for age, sex, maternal education(low/high), weekend, season (April-September / October-March) and the other light exposure metrics.

**Supplementary Table ST3.** Results of the beta mixed effects regression analysis on sleep efficiency based on total time in bed according to Actigraph.

|  | Minimally adjusted  Exp Coefficients (95% CI) | Sleep efficiency (%)  Fully adjusted  Exp Coefficients (95% CI) | Mutually adjusted  Exp Coefficients (95% CI) |
| --- | --- | --- | --- |
| Timing bright light morning (h before sleep) | 1.00 [0.99, 1.01] | 1.00 [0.99, 1.01] | 1.01 [0.99, 1.03] |
| Timing bright light afternoon (h before sleep) | 1.01 [1.00, 1.02] | 1.02 [1.00, 1.03] | 1.02 [1.00, 1.04] |
| Duration of bright light (hours) |  |  |  |
| **Below median:** < 0.9 | Reference group | Reference group | Reference group |
| **Median-90th perc.:** 0.9 to 3.0 | 0.98 [0.92, 1.04] | 0.99 [0.93, 1.05] | 0.97 [0.90, 1.05] |
| **>90th percentile:** > 3.0 | 0.97 [0.89, 1.06] | 1.00 [0.91, 1.10] | 1.00 [0.89, 1.13] |
|  |  |  | *p-trend=0.90* |
| Average light intensity (lux) before going to bed |  |  |  |
| **Below median:** < 12 | Reference group | Reference group | Reference group |
| **Median-90th perc.:** 12 to 79 | 0.96 [0.91, 1.01] | 0.95 [0.90, 1.01] | 0.93 [0.86, 1.00] |
| **>90th percentile:** > 80 | 0.91 [0.84, 1.00] | **0.91 [0.83, 0.99]** | 0.96 [0.86, 1.09] |
|  |  |  | *p-trend=0.97* |
| Average light intensity (lux) when in bed |  |  |  |
| **Below median:** < 0.07 | Reference group | Reference group | Reference group |
| **Median-90th perc.:** 0.07 to 0.4 | 0.95 [0.88, 1.02] | 0.95 [0.88, 1.03] | 0.95 [0.88, 1.04] |
| **>90th percentile:** > 0.4 | 0.90 [0.80, 1.01] | 0.91 [0.81, 1.02] | 0.88 [0.77, 1.00] |
|  |  |  | *p-trend=0.06* |

**Sleep efficiency** **(%)**: the sleep duration divided by the total time in bed, both measured by the Actigraph (difference between onset and waking time); **The timing of** **bright light in the morning**: the amount of hours between the first time of bright light in the morning and going to sleep; **The timing of bright light in the afternoon**: the amount of hours between the last time of bright light in the afternoon and going to sleep; **Bright light duration**: the duration of bright daylight exposure with a cut-off at 1000 lux (h); **Average light intensity before going to bed**: the average light intensity in the hour before going to bed (lux); **Average light intensity when in bed**: the average light intensity during the darkest part of the night (00:12 – 03:10) (lux); **Minimally adjusted:** Model adjusted for age and sex; **Fully adjusted:** Model adjusted for age, sex, maternal education (low/high), weekend night (yes/no) and season (April-September / October-March); **Mutually adjusted:** Model adjusted for age, sex, maternal education(low/high), weekend, season (April-September / October-March) and the other light exposure metrics.

**Supplementary Table ST4**. Results of the logistic mixed effects regression analysis on sleep onset delay.

|  | Minimally adjusted  OR (95% CI) | Sleep onset delay (min)   Fully adjusted  OR (95% CI) | Mutually adjusted  OR (95% CI) |
| --- | --- | --- | --- |
| Timing bright light morning (h before sleep) | 0.96 [0.91, 1.00] | 0.96 [0.91, 1.00] | **0.88 [0.80, 0.97]** |
| Timing bright light afternoon (h before sleep) | 0.94 [0.87, 1.00] | 0.93 [0.86, 1.00] | 1.04 [0.94, 1.15] |
| Duration of bright light (hours) |  |  |  |
| **Below median:** < 0.9 | Reference group | Reference group | Reference group |
| **Median-90th perc.:** 0.9 to 3.0 | 0.91 [0.66, 1.26] | 0.85 [0.61, 1.19] | 0.98 [0.62, 1.57] |
| **>90th percentile:** > 3.0 | **0.52 [0.30, 0.92]** | **0.45 [0.25, 0.81]** | **0.40 [0.19, 0.87]** |
|  |  |  | ***p trend=0.02*** |
| Average light intensity (lux) before going to bed |  |  |  |
| **Below median:** < 12 | Reference group | Reference group | Reference group |
| **Median-90th perc.:** 12 to 79 | 1.25 [0.90, 1.72] | 1.26 [0.91, 1.74] | 1.27 [0.83, 1.94] |
| **>90th percentile:** > 80 | **2.41 [1.49, 3.91]** | **2.45 [1.49, 4.02]** | **4.02 [2.09, 7.73]** |
|  |  |  | ***p trend < 0.001*** |
| Average light intensity (lux) when in bed |  |  |  |
| **Below median:** < 0.07 | Reference group | Reference group | Reference group |
| **Median-90th perc.:** 0.07 to 0.4 | 1.02 [0.68, 1.52] | 1.00 [0.67, 1.49] | 1.03 [0.65, 1.62] |
| **>90th percentile:** > 0.4 | 0.87 [0.46, 1.65] | 0.82 [0.43, 1.56] | 1.30 [0.65, 2.62] |
|  |  |  | *p trend=0.50* |

**Sleep onset delay (min)**: the difference between sleep onset as estimated by the Actigraph and estimated by the sleep logs; **The timing of** **bright light in the morning**: the amount of hours between the first time of bright light in the morning and going to sleep; **The timing of bright light in the afternoon**: the amount of hours between the last time of bright light in the afternoon and going to sleep; **Bright light duration**: the duration of bright daylight exposure with a cut-off at 1000 lux (h); **Average light intensity before going to bed**: the average light intensity in the hour before going to bed (lux); **Average light intensity when in bed**: the average light intensity during the darkest part of the night (00:12 – 03:10) (lux); **Minimally adjusted:** Model adjusted for age and sex; **Fully adjusted:** Model adjusted for age, sex, maternal education (low/high), weekend night (yes/no) and season (April-September / October-March); **Mutually adjusted:** Model adjusted for age, sex, maternal education(low/high), weekend, season (April-September / October-March) and the other light exposure metrics.

**Supplementary Table ST5.** Results of the beta mixed effects regression analysis on sleep efficiency according to the sleep log (sensitivity analysis).

|  | Minimally adjusted  Exp Coefficients (95% CI) | Sleep efficiency (%)  Fully adjusted  Exp Coefficients (95% CI) | Mutually adjusted  Exp Coefficients (95% CI) |
| --- | --- | --- | --- |
| Timing bright light morning (h before sleep) | 1.00 [1.00, 1.01] | 1.00 [0.99, 1.01] | **1.02 [1.01, 1.04]** |
| Timing bright light afternoon (h before sleep) | 1.01 [1.00, 1.02] | 1.01 [1.00, 1.02] | 1.00 [0.99 1.02] |
| Duration of bright light (hours) |  |  |  |
| **Below median:** < 0.9 | Reference group | Reference group | Reference group |
| **Median-90th perc.:** 0.9 to 3.0 | 0.99 [0.95, 1.05] | 1.00 [0.95, 1.05] | 0.96 [0.90, 1.02] |
| **>90th percentile:** > 3.0 | 1.02 [0.95, 1.11] | 1.05 [0.98, 1.14] | 1.05 [0.94, 1.16] |
|  |  |  | *p-trend =0.61* |
| Average light intensity (lux) before going to bed |  |  |  |
| **Below median:** < 12 | Reference group | Reference group | Reference group |
| **Median-90th perc.:** 12 to 79 | 0.99 [0.94, 1.03] | 0.98 [0.94, 1.03] | 0.97 [0.91, 1.03] |
| **>90th percentile:** > 80 | 0.93 [0.86, 1.00] | 0.93 [0.86, 1.00] | 0.92 [0.83, 1.02] |
|  |  |  | *p-trend =0.20* |
| Average light intensity (lux) when in bed |  |  |  |
| **Below median:** < 0.07 | Reference group | Reference group | Reference group |
| **Median-90th perc.:** 0.07 to 0.4 | 0.97 [0.91, 1.03] | 0.97 [0.91, 1.04] | 1.00 [0.93, 1.07] |
| **>90th percentile:** > 0.4 | **0.88 [0.80, 0.98]** | **0.89 [0.80, 0.99]** | **0.87 [0.78, 0.97]** |
|  |  |  | ***p-trend =0.009*** |

**Sleep efficiency** **(%)**: the sleep duration divided by the total time in bed based on the sleep logs (difference between onset and waking time); **The timing of** **bright light in the morning**: the amount of hours between the first time of bright light in the morning and going to sleep; **The timing of bright light in the afternoon**: the amount of hours between the last time of bright light in the afternoon and going to sleep; **Bright light duration**: the duration of bright daylight exposure with a cut-off at 1000 lux (h); **Average light intensity before going to bed**: the average light intensity in the hour before going to bed (lux); **Average light intensity when in bed**: the average light intensity during the darkest part of the night (00:12 – 03:10) (lux): **Minimally adjusted:** Model adjusted for age and sex; **Fully adjusted:** Model adjusted for age, sex, maternal education (low/high), weekend night (yes/no) and season (April-September / October-March); **Mutually adjusted:** Model adjusted for age, sex, maternal education(low/high), weekend, season (April-September / October-March) and the other light exposure metrics.
